# Effects of human chorionic gonadotropin on the last day of a 5-day CIDR Synch protocol and 5 days later on circulating progesterone concentrations and luteal area in Holstein heifers

**DOI:** 10.3168/jdsc.2022-0220

**Published:** 2022-06-17

**Authors:** Marcelo S. El Azzi, Teresita Valdes-Arciniega, Everardo Anta-Galvan, Iago M.R. Leão, Rodrigo V. Sala, Meliton Fosado, José C. de Souza, João Paulo N. Martins

**Affiliations:** 1 Department of Medical Sciences, School of Veterinary Medicine, University of Wisconsin-Madison 53706; 2 Faculdade de Zootecnia e Medicina Veterinária, Universidade Federal de Lavras, Lavras MG 37200-900 Brazil; 3 ST Genetics, South Charleston, OH 45368; 4 ST Genetics, Kewaskum, WI 53040

## Abstract

The objective was to determine the effect of replacing GnRH with human chorionic gonadotropin (hCG) on the last day of a 5-d CIDR Synch protocol (d 0) and inducing accessory corpus luteum (CL) formation with hCG 5 d later (d 5) on serum progesterone (P4) concentrations and luteal dynamics in dairy heifers. Holstein heifers (n = 207) were synchronized with a 5-d controlled internal drug release (CIDR) Synch protocol (d −8: used CIDR inserted; d −3: CIDR removed and PGF_2α_). Heifers were randomly assigned to 1 of 4 treatments on d 0: control (G; n = 55), H (n = 50), GH (n = 53), and HH (n = 49). Heifers in G were treated with 100 μg of GnRH on d 0, while H heifers received hCG (3,300 IU) on d 0. Heifers enrolled in GH were treated with GnRH on d 0 and hCG on d 5, while HH received hCG on d 0 and 5. Ovaries were scanned by ultrasound on d 0, 5, and 12, and blood was collected on d 0, 5, 7, and 12. Heifers that ovulated before or after the hCG or GnRH d 0 treatment and had P4 ≤ 0.50 ng/mL on d 0 were considered as synchronized. Overall protocol synchronization response was 93.8%, with no differences among treatments. Only synchronized heifers (n = 193) were included in the analyses of luteal dynamics after d 0. Serum P4 concentration and original CL luteal area on d 5 in heifers treated with hCG on d 0 (H + HH) were greater than in heifers treated with GnRH on d 0 (G + GH). Almost all heifers treated with hCG on d 5 had ≥2 CL on d 12 (98.6%). Ovulatory response for d 5 hCG treatment did not differ for GH versus HH (97.2 vs. 94.7%). Heifers in HH had the highest serum P4 on d 7, and G had the lowest serum P4 on d 7 and 12. In contrast, serum P4 on d 7 did not differ for H versus GH. On d 12, serum P4 and total luteal area were not different for GH versus HH. In summary, heifers that received hCG on d 0 had a larger total luteal area and greater serum P4 concentration on d 5 than heifers treated with GnRH on d 0. Moreover, hCG on d 5 promoted a greater proportion of heifers with ≥2 CL on d 12 and a larger luteal area of the original CL, which resulted in a larger total luteal area on d 12. The HH treatment successfully increased serum P4 concentrations in heifers on d 7 compared with the other treatments.

## Highlights

• The hCG on the last day of a 5-day CIDR Synch (day 0) enhanced original CL area on day 5.
• The hCG versus GnRH on day 0 increased serum P4 on day 5, 7, and 12.
• The hCG on day 5 induced accessory CL formation and increased serum P4 on day 7 and 12.
• The hCG on day 5 augmented original CL area on day 12 compared with no hCG on day 5.
• The hCG on day 0 and 5 (HH) increased serum P4 on day 7 compared with the other treatments.

## Graphical Abstract

**Figure:**
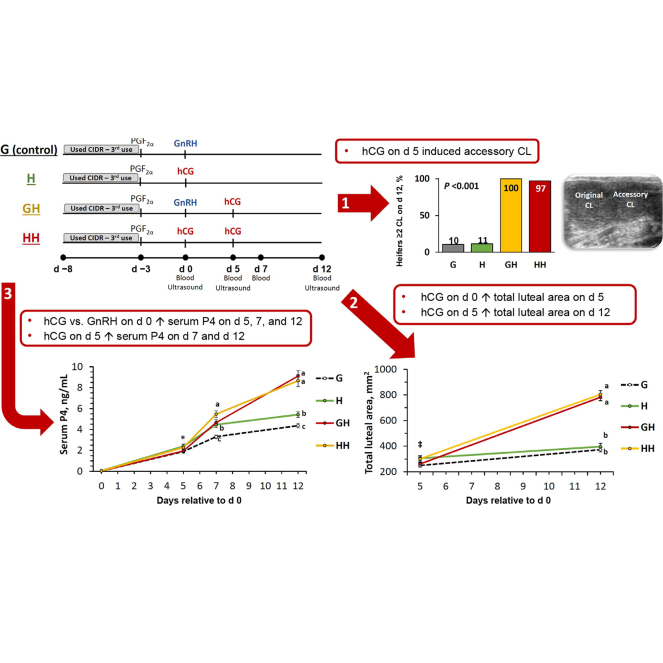
**Summary:** Our objective was to determine the effect of replacing gonadotropin-releasing hormone (GnRH) with human chorionic gonadotropin (hCG) on the last day of a 5-day controlled internal drug release (CIDR) Synch (day 0) and inducing corpus luteum (CL) formation with hCG on day 5 on serum progesterone (P4) concentration and luteal dynamics in dairy heifers. Heifers receiving hCG on day 0 had a larger luteal area and greater serum P4 concentration on day 5 than heifers receiving GnRH on day 0. Heifers that received hCG on day 0 and 5 had greater serum P4 concentrations on day 7 than the heifers in the other treatments. In addition, hCG on day 5 promoted a greater proportion of heifers with ≥2 CL on day 12 and a larger luteal area of the original CL, which resulted in a larger total luteal area on day 12.

Optimizing circulating progesterone (**P4**) concentration during early embryo development is fundamental for the establishment of pregnancy. Previous studies used different strategies to increase circulating P4 concentrations during early embryo development and improve fertility of recipient cows and heifers (Rizos et al., 2012; Monteiro et al., 2015; Steichen and Larson, 2019). However, the use of those strategies has yielded inconsistent pregnancy results after embryo transfer (**ET**). One of the strategies tested was to induce an accessory corpus luteum (**CL**) with GnRH or human chorionic gonadotropin (**hCG**) during the first follicular wave (Niles et al., 2019; García-Guerra et al., 2020). The fertility results from those studies may not be consistent because of a delayed increase in circulating P4 concentrations. Recently, Garcia-Ispierto et al. (2021) tested the use of hCG (3,000 IU) replacing GnRH (100 µg) at the last treatment of a 5-d controlled internal drug release (**CIDR**) Synch protocol in recipient lactating Holstein cows. Although the study sample size was small (n = 120), hCG increased the odds (3.3) for pregnancy at 28 d compared with GnRH. In that study, the effect of hCG on luteal development and circulating P4 concentrations during the estrous cycle was not determined. The use of hCG instead of GnRH at the last treatment of synchronization programs may induce the formation of a CL with greater P4 synthesis capability and greater circulating P4 concentrations earlier, improving fertility of recipient heifers or cows.

Therefore, our study aimed to determine the effect of 3,300 IU of hCG administered on the last day of a 5-d CIDR Synch program (d 0) with or without an extra administration of hCG 5 d later (d 5) on luteal development and circulating P4 concentrations in metestrus and diestrus of Holstein heifers. The primary hypothesis was that heifers treated with hCG on d 0 would have greater total luteal area and serum P4 concentration during metestrus and diestrus compared with heifers treated with GnRH on d 0. The second hypothesis was that heifers treated with hCG on d 0 and 5 would have greater total luteal area and serum P4 concentrations during diestrus than those treated with hCG only on d 0 or d 5.

All animal handling and experimental procedures were approved by the Animal Care and Use Committee at the University of Wisconsin-Madison (#A006300). This experiment was conducted in a commercial dairy farm in Wisconsin from September to November of 2019. A total of 207 recipient Holstein heifers between 11 and 22 mo old (mean ± SD = 15.0 ± 2.8; median = 14.5) with mean BCS ± SD of 3.3 ± 0.2 [1 = emaciated, 5 = obese (Ferguson et al., 1994)] were enrolled in the study. Heifers were housed in a freestall barn, bedded with dried manure solids, free access to water, and fed once daily with a TMR formulated to meet or exceed the nutritional requirements of Holstein heifers weighing 360 kg and gaining 0.8 kg/d (NRC, 2001).

Weekly cohorts of heifers were synchronized with a 5-d CIDR Synch protocol using a CIDR (1.38 g of P4, Eazi-Breed CIDR, Zoetis) previously used twice for 5 d each time. After each use, CIDR inserts were individually washed with water and soaked in a solution of 2% chlorhexidine diacetate (Novalsan Solution, Zoetis) for 15 min, as described by Sala et al. (2020). The 5-d CIDR Synch protocol consisted of intravaginal insertion of a CIDR on d −8 and withdrawal 5 d later, followed by the administration of PGF_2α_ (500 mg of cloprostenol i.m.; Estroplan, Parnell) on d −3. Three days later, on d 0, heifers were randomly assigned to receive 1 of 4 treatments: control (**G**), **H**, **GH**, and **HH**. Heifers in G (n = 53) were only treated with GnRH (100 µg i.m.; Gonabreed, Parnell) on d 0, whereas heifers in the H group (n = 49) were treated with hCG (3,300 IU i.m.; Chorulon, Merck Animal Health) on d 0. Heifers assigned to GH (n = 52) were treated with GnRH on d 0 and hCG 5 d later (d 5). In the HH treatment, heifers (n = 48) received hCG on d 0 and 5.

A trained technician scanned ovaries by transrectal ultrasonography using a Easi-scan:Go ultrasound unit with a 7.5-MHz linear array probe (IMV Imaging) paired to an iPad mini 4 (Apple Inc.). Ovarian ultrasonography examinations were performed on d 0 (n = 202), d 5 (n = 194), and d 12 (n = 159). Ultrasound videos were recorded with the IMV Go Scan application (version 3.70; BCF Technology Ltd.) using an iPad mini. Ovarian structures were measured using a video metrics analysis software (Kinovea 0.8.15; Kinovea.org), and calipers were calibrated with background gridlines (size = 10 mm). Follicle ≥7 mm, CL, and CL cavity (if present) mean diameters were calculated by the average of the height and width. Total CL and CL cavity area were calculated by the equation 0.5 height × 0.5 width × π (Martins et al., 2011). The total luteal area of each CL was calculated by subtracting the CL cavity area from the total CL area.

Ovulation was defined upon detection of a new CL in the subsequent scan (d 5 or 12) in the same location of a follicle with a diameter ≥9 mm from the previous scan (d 0 or 5). Heifers missing scans on d 0 (n = 5) or d 5 (n = 13) were considered to ovulate in response to the d 0 GnRH or hCG treatments when P4 was ≤0.50 ng/mL on d 0 and ≥1 ng/mL on d 7. Heifers were considered to have ovulated before GnRH or hCG treatment on d 0 when they had P4 ≤0.50 ng/mL on d 0, no ≥9 mm antral-follicle present on d 0, and the presence of a new CL ≥14 mm on d 5 that increased in size by d 12. Heifers that ovulated before or after the hCG or GnRH d 0 treatment and had P4 ≤0.50 ng/mL on d 0 were considered as synchronized.

Blood samples were collected by puncture of the coccygeal vein or artery into vacuum tubes (Vacuette Z serum clot activator, Greiner Bio-One International GmbH) on d 0 (n = 195), d 5 (n = 194), d 7 (n = 193), and d 12 (n = 164). Blood samples were stored in a cooler with ice, transported to the laboratory, and processed within 8 h after collection. Serum was separated by centrifugation at 2,000 × *g* for 10 min at 4°C and stored at −20°C for later hormonal assays. Serum P4 concentrations were measured using a solid-phase RIA kit (ImmuChem coated tube Progesterone; MP Biomedicals). Mean assay sensitivity was 0.02 ng/mL. Intra- and interassay coefficients of variation were 6.0% and 1.75%, respectively.

This study used a complete randomized experimental design. A priori power analysis was performed in version 3.1.9.6 of the G*Power analysis software (Faul et al., 2009). The required sample size was calculated using *t*-test, 2-tail, α = 0.05, power = 0.95, and 0.80 of effect size |ρ|. A total of 42 heifers per treatment was required to find a difference in serum P4 concentrations between 2 independent means of 2.00 versus 2.40 ng/mL using SD within each group of 0.50.

All statistical analyses were performed using version 9.4 of SAS (SAS Institute Inc.). Binary outcomes such as ovulatory responses, ovulation before and after d 0, ovulation on d 5, the proportion of synchronized heifers, and CL presence were analyzed by generalized linear mixed models considering a binary distribution and a logit link function using the GLIMMIX procedure. Treatment was considered as a fixed effect in the model. Continuous outcomes such as follicle diameter, primary and accessory CL area, total luteal area, and circulating P4 concentrations were analyzed by ANOVA using the MIXED procedure. The model included treatment, days, and interaction as fixed effects. For the analysis of the effect of d 0 treatment (GnRH or hCG) and time of ovulation (before or after d 0) on mean luteal area of original CL and serum P4 concentrations, the model included the fixed effects of d 0 treatment, time of ovulation, and interaction d 0 treatment × time of ovulation. The LSMeans statement was used to detect differences in d 0 treatments (GnRH vs. hCG) within each time of ovulation (before or after d 0). Normality and homoscedasticity of residuals were evaluated by Studentized residual plots for each variable after fitting the model using the residual option of the MIXED procedure.

The effect of treatment on total luteal area and circulating P4 concentrations over time was analyzed using the MIXED procedure with the REPEATED statement with cow (treatment) specified in the SUBJECT option. The unstructured covariance structure was used for these analyses. Treatment, day, and treatment × day interaction were included as fixed effects in the model. For the analysis of circulating P4 concentrations over time, P4 concentrations were log-transformed to fulfill normality assumptions. Actual means ± standard error of the mean of the data were presented for clarity. Differences among treatments were considered significant when *P* ≤ 0.05, whereas *P* > 0.05 and *P* ≤ 0.10 were considered a tendency. Data were presented as means ± standard error of the mean for continuous outcomes and as proportions for binary outcomes.

Follicular and luteal parameters evaluated in response to the last 2 hormone treatments of the 5-d CIDR Synch protocol did not differ (*P* > 0.52) among treatments (Table 1). Overall proportion of heifers with P4 ≤0.50 ng/mL on d 0 was 95.2%. Overall mean ovulatory response before or after d 0 was 98.1% and did not differ among treatments (Table 1) or between hCG and GnRH (Table 2) on d 0. Approximately half (46.4%) of the heifers submitted to the 5-d CIDR Synch program in our study ovulated before the treatment with GnRH or hCG on d 0. These heifers were most likely on d 1 or 2 of metestrus, characterized by serum P4 concentrations <0.50 ng/mL and absence of a CL >14 mm in diameter on d 0. In a previous study using a similar 5-d CIDR Synch protocol with a third or fourth use CIDR, time of ovulation averaged ~86 h after CIDR removal (Sala et al., 2020). In that study, 33.9% of the heifers ovulated before d 0 (personal communication, Rodrigo Sala, ST Genetics, South Charleston, OH), which is lower than in the present study. Heifers with a dominant follicle in an advanced development stage at d −3 with complete luteolysis (P4 ≤ 0.50 ng/mL) before or after PGF_2α_ are more likely to ovulate before d 0. Using GnRH at d −8 might have decreased the proportion of heifers ovulating before d 0 because fewer heifers would be in later stages of follicular development at d −3 (day of PGF_2α_). However, in a previous study, GnRH at the initiation of a 5d-CIDR Synch protocol (d −8) with a new CIDR did not increase the proportion of heifers in estrus on d 0 (Lima et al., 2013).

**Table 1:** Effect of treatment^1^ on follicular and luteal parameters from d 0 to 12 in Holstein heifers that received the 5-d controlled internal drug release (CIDR) Synch protocol

Item	G	H	GH	HH	*P*-value
Ovulation before d 0, % (n/n)	49.1 (26/53)	42.9 (21/49)	51.9 (27/52)	41.7 (20/48)	0.69
Ovulation after d 0, % (n/n)	49.1 (26/53)	53.1 (26/49)	42.3 (22/52)	56.3 (27/48)	0.54
Ovulation before and after d 0, % (n/n)	0 (0/53)	2.0 (1/49)	1.9 (1/52)	2.1 (1/48)	0.99
Total ovulation, % (n/n)	98.2 (52/53)	98.1 (48/49)	96.1 (50/52)	100 (48/48)	0.93
Largest ovulatory follicle diameter on d 0,^2^ mm ± SEM	13.3 ± 0.4	12.5 ± 0.3	12.6 ± 0.5	13.1 ± 0.4	0.53
Heifers with P4 ≤0.50 ng/mL on d 0, % (n/n)	94.6 (52/55)	96.0 (48/50)	96.3 (52/53)	98.0 (48/49)	0.85
Heifers synchronized,^3^ % (n/n)	92.7 (51/55)	94.0 (47/50)	94.5 (51/53)	93.9 (46/49)	0.99
Heifers with n ≥2 CL on d 5,^4^ % (n/n)	3.9 (2/51)	8.5 (4/47)	2.0 (1/50)	10.9 (5/46)	0.31
Ovulation between d 5 and 12,^5^ % (n/n)	4.9^b^ (2/41)	4.6^b^ (2/44)	97.2^a^ (35/36)	94.7^a^ (36/38)	<0.001
Largest ovulatory follicle diameter on d 5,^6^ mm ± SEM	9.3 ± 0.1^b^	10.9 ± 0.9^b^	11.5 ± 0.2^a^	11.8 ± 0.3^a^	<0.001
Double ovulation between d 5 and 12,^6^ % (n/n)	0 (0/2)	0 (0/2)	8.6 (3/35)	19.4 (7/36)	0.65
Heifers with n ≥2 CL on d 12,^5^ % (n/n)	9.8^b^ (4/41)	11.4^b^ (5/44)	100^a^ (36/36)	97.4^a^ (37/38)	<0.001
Heifers with ipsilateral accessory CL on d 12,^6^ % (n/n)	50 (1/2)	50 (1/2)	48.6 (17/35)	52.7 (19/36)	0.99

a,b Means with different superscripts within a row differ (*P* < 0.05).

1 Treatments: G = GnRH on d 0; H = human chorionic gonadotropin (hCG) on d 0; GH = GnRH on d 0 and hCG on d 5; HH = hCG on d 0 and 5.

2 Only heifers with ovulation after d 0 GnRH or hCG treatment.

3 Heifers with progesterone (P4) ≤0.50 ng/mL on d 0 and ovulation before or after the d 0 treatment (GnRH or hCG) were considered synchronized.

4 Only synchronized heifers. CL = corpus luteum.

5 Only synchronized heifers with scans on d 5 and 12 (n = 159).

6 Only synchronized heifers with ovulation between d 5 and 12 (n = 75).

**Table 2:** Effect of d 0 treatment with GnRH versus human chorionic gonadotropin (hCG; treatments^1^ combined) on follicular and luteal parameters from d 0 to d 5 in Holstein heifers that received the 5-d CIDR Synch protocol

Item	GnRH on d 0 (G + GH)	hCG on d 0 (H + HH)	*P*-value
Ovulation before d 0, % (n/n)	50.5 (53/105)	42.3 (41/97)	0.25
Ovulation after d 0, % (n/n)	45.7 (48/105)	54.6 (53/97)	0.21
Ovulation before and after d 0, % (n/n)	0.9 (1/105)	2.1 (2/97)	0.50
Total ovulation, % (n/n)	97.1 (102/105)	99.0 (96/97)	0.37
Largest ovulatory follicle diameter on d 0,^2^ mm ± SEM	12.9 ± 0.3	12.8 ± 0.3	0.73
Heifers with P4 ≤0.50 ng/mL on d 0, % (n/n)	95.4 (103/108)	97.0 (96/99)	0.55
Heifers synchronized,^3^ % (n/n)	93.5 (101/108)	94.0 (93/99)	0.90
Heifers with n ≥2 CL on d 5,^4^ % (n/n)	3.0 (3/101)	9.7 (9/93)	0.06
Serum P4 on d 5, ng/mL ± SEM	1.91 ± 0.10	2.37 ± 0.14	0.007
Luteal area of original CL on d 5,^5^ mm^2^ ± SEM	260 ± 9	306 ± 13	0.002

1 Treatments: G = GnRH on d 0; H = hCG on d 0; GH = GnRH on d 0 and hCG on d 5; HH = hCG on d 0 and 5.

2 Only heifers with ovulation after d 0 GnRH or hCG treatment.

3 Heifers with progesterone (P4) ≤0.50 ng/mL on d 0 and ovulation before or after the d 0 treatment (GnRH or hCG) were considered synchronized.

4 Only synchronized heifers. CL = corpus luteum.

5 Only heifers with only one CL on d 5 were used in this analysis.

Furthermore, hCG treatment on d 0 (H + HH) did not affect (*P* = 0.90) synchronization parameters in comparison to GnRH (G + GH; Table 2). The overall synchronization response for the 5-d CIDR Synch program was 93.8%. The synchronization response in the present experiment indicates the proportion of heifers that would be on d 0, 1, or 2 of the estrous cycle on study d 0 and most likely to be used as recipients for ET on study d 6 and 7. Sala et al. (2020), using a similar protocol with third use CIDR in Holstein heifers, also found a similar ovulatory response (97%) and an ET utilization ratio (transferred/treated) of 93.3%. In the same study, pregnancy per ET did not differ between heifers receiving ET 7 ± 1 d after detection of estrus and after a 5-d CIDR Synch protocol with a new or second use CIDR (Sala et al., 2020).

Treatment on d 0 (hCG vs. GnRH) affected (*P* < 0.01) circulating P4 concentrations at d 5. Heifers treated with hCG on d 0 (H + HH) had greater (*P* < 0.01) serum P4 and original CL area on d 5 than heifers treated with GnRH on d 0 (G + GH; Table 2). Moreover, treatment with hCG on d 0 also tended (*P* = 0.06) to increase the proportion of heifers with ≥2 CL at d 5 (Table 2). The increase in original CL size supports the increase in serum P4 on d 5 for heifers treated with hCG on d 0.

Time of ovulation relative to d 0 treatment had an effect (*P* < 0.001) on circulating P4 concentrations and total luteal area on d 5. Heifers ovulating before d 0 had greater (*P* < 0.001) serum P4 concentration (3.60 ± 0.15 vs. 2.06 ± 0.13 ng/mL, respectively) and total luteal area (313 ± 10 vs. 246 ± 10 mm^2^, respectively) on d 5 compared with heifers that ovulated after d 0. These results were expected because heifers with ovulation before d 0 would be in a later stage of the estrous cycle (1 or 2 d later) with a more mature CL on d 5, synthesizing more P4, compared with heifers with ovulation after d 0. No interaction between ovulation time relative to d 0 and d 0 treatment was found on d 5 total luteal area (*P* = 0.50) and serum P4 concentration (*P* = 0.53). These results suggest that exogenous administration of 3,300 IU of hCG had a greater steroidogenic effect than the GnRH-induced LH surge not only during early CL development (after ovulation) but also just before ovulation and formation of the CL. It is not clear whether this effect occurred in the pre-ovulatory follicle cells, luteal cells after ovulation, or both. We speculate that hCG may have induced faster or increased luteinization of granulosa cells or that hCG, due to its greater half-life than LH, acts for a longer period on progesterone synthesis after the transformation of follicular cells into luteal cells (Schmitt et al., 1996).

Ovulation to hCG on d 5 did not differ between GH and HH (Table 1). Overall mean ovulatory response to hCG treatment on d 5 was 96% (71/74), which was similar to the overall ovulation to d 0 hCG (99.0%) and GnRH (97.1%; Table 2). About 97% of heifers treated with hCG on d 5 had ≥2 CL on d 12, indicating that 3,300 IU of hCG on d 5 of the estrous cycle is highly effective in inducing ovulation and formation of an accessory CL in Holstein heifers. In contrast, fewer (*P* < 0.001) heifers (11.8%) not treated with hCG on d 5 had ≥2 CL at d 12 due to double ovulation to d 0 treatment (GnRH or hCG). In addition to the induction of an accessory CL, hCG treatment on d 5 also increased (*P* < 0.001) the luteal area of the original CL on d 12 for heifers with a single CL on d 5 (no hCG on d 5: 369 ± 15 vs. hCG on d 5: 464 ± 20). In a different study using 3,300 IU of hCG on d 7 of the estrous cycle, an increase in original CL volume during diestrus was observed (Cunha et al., 2022). This effect of hCG on original CL may have been due to the prolonged half-life of hCG (Yen et al., 1968) and high affinity for LH receptors (Schmitt et al., 1996). Treatments not including hCG on d 5 (G and H) had a smaller original CL area at d 12 than treatments with hCG on d 5 (GH and HH; Figure 1). In addition, the luteal area of the original CL at d 12 did not differ between GH and HH (Figure 1). These results suggest that the effect of hCG on original CL appears to be transient, and the difference in original CL area found between H and G was not maintained from d 5 to d 12. Furthermore, accessory CL area did not differ between GH and HH on d 12 (*P* = 0.11; Figure 1).

**Figure 1:**
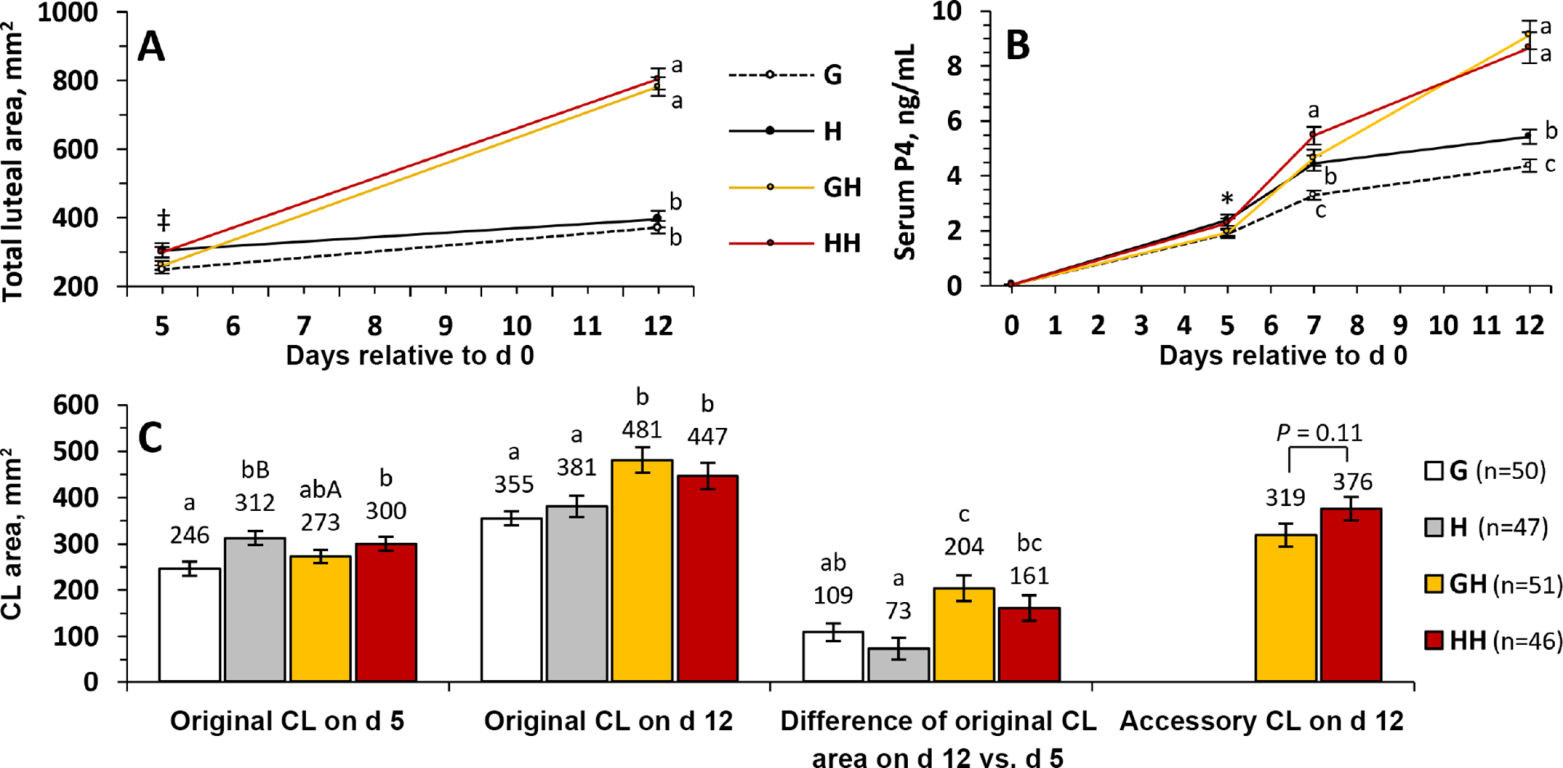
Effect of treatment on (A) total luteal area and (B) serum progesterone (P4) concentrations over time after d 0. Also, effect of treatment on (C) original corpus luteum (CL) area on d 5 and d 12, the difference of original CL area on d 12 versus d 5 and on accessory CL area on d 12. ‡Symbol indicates difference between G and HH (*P* = 0.01) on d 5. *Asterisk indicates differences between G and H on d 5 (*P* = 0.05). Means with different letters (a–c) differ (*P* < 0.05) within a day or CL type. Means with different letters (A,B) tend to differ (*P* < 0.10) within a CL type. Original CL analysis: treatment *P* < 0.002; day *P* < 0.001; and treatment × day *P* < 0.001. Treatments: G = GnRH on d 0; H = human chorionic gonadotropin (hCG) on d 0; GH = GnRH on d 0 and hCG on d 5; HH = hCG on d 0 and 5.

Treatment also affected circulating P4 concentrations on d 7 and 12 (Figure 1). Heifers in G had the lowest mean serum P4 concentrations on d 7 and 12 compared with the other treatments (Figure 1). Mean serum P4 at d 7 did not differ between H and GH (*P* = 0.83). Heifers treated with HH had the highest serum P4 at d 7 among treatments, but at d 12, serum P4 was similar between GH and HH. The greater mean serum P4 at d 7 and 12 for H compared with G suggests that hCG has a prolonged effect on serum P4 compared with GnRH-induced LH surge. This effect of d 0 hCG on serum P4 does not appear to be only the effect of the increase in the total luteal area because the luteal area at d 12 did not differ between H and G. The effect of hCG at d 0 on serum P4 at d 12 was not evident when heifers received another hCG treatment at d 5, indicating that the major driver for the increase in serum P4 at d 12 was the formation of an accessory CL induced by hCG at d 5.

A recent study that used 1,500 IU of hCG on d 5 in cross-bred heifers found an effect of accessory CL side relative to original CL (ipsilateral vs. contralateral) on original CL area and circulating P4 concentrations (Hazano et al., 2020). In that study, contralaterally induced CL had greater diameter and serum P4 concentration on d 7 and 14 (*P* ≤ 0.05) in comparison with ipsilaterally induced CL (Hazano et al., 2020). In contrast, in the present study, side of accessory CL relative to original CL did not affect original CL luteal area (*P* = 0.20; ipsilateral: 431 ± 28 vs. contralateral: 480 ± 25 mm^2^), or accessory CL luteal area (*P* = 0.64; ipsilateral: 357 ± 24 vs. contralateral: 340 ± 27 mm^2^) on d 12. Moreover, total luteal area (*P* = 0.43; ipsilateral: 787 ± 26 vs. contralateral: 820 ± 31 mm^2^) and serum P4 concentrations (*P* = 0.78; ipsilateral: 11.74 ± 0.66 vs. contralateral: 11.47 ± 0.69 ng/mL) on d 12 did not differ for heifers with ipsilateral and contralateral accessory CL.

In summary, the administration of hCG on the last day of a 5-d CIDR Synch program increased circulating P4 concentrations and luteal area 5 d later compared with heifers treated only with GnRH independent of the ovulation time relative to d 0 treatment. The treatments with hCG on d 5 induced accessory CL formation and increased total luteal area on d 12 and serum P4 concentrations on d 7 and 12. The HH treatment successfully increased serum P4 concentrations in heifers on d 7 compared with the other treatments. If applied in ET programs in recipient heifers, this strategy may potentially increase pregnancy. Future research is warranted to determine the effect of the presented treatments on pregnancy per ET.

## References

[bib1] Cunha T.O., Statz L.R., Domingues R.R., Andrade J.P.N., Wiltbank M.C., Martins J.P.N. (2022). Accessory corpus luteum induced by human chorionic gonadotropin on day 7 or days 7 and 13 of the estrous cycle affected follicular and luteal dynamics and luteolysis in lactating Holstein cows. J. Dairy Sci..

[bib2] Faul F., Erdfelder E., Buchner A., Lang A.-G. (2009). Statistical power analyses using G*Power 3.1: Tests for correlation and regression analyses. Behav. Res. Methods.

[bib3] Ferguson J.D., Galligan D.T., Thomsen N. (1994). Principal descriptors of body condition score in Holstein cows. J. Dairy Sci..

[bib4] García-Guerra A., Sala R.V., Carrenho-Sala L., Baez G.M., Motta J.C.L., Fosado M., Moreno J.F., Wiltbank M.C. (2020). Postovulatory treatment with GnRH on day 5 reduces pregnancy loss in recipients receiving an in vitro produced expanded blastocyst. Theriogenology.

[bib5] Garcia-Ispierto I., Llobera-Balcells M., López-Gatius F. (2021). Inducing ovulation with human chorionic gonadotrophin improves the pregnancy rate in lactating dairy cows receiving an in vitro-produced embryo. Reprod. Domest. Anim..

[bib6] Hazano K., Haneda S., Kayano M., Miura R., Matsui M. (2020). Effects of hcg administration on corpus luteum development and plasma sex steroid hormone concentration in beef heifers differ according to the locational relationships of the original corpus luteum and the first-wave dominant follicle. J. Vet. Med. Sci..

[bib7] Lima F.S., Ribeiro E.S., Bisinotto R.S., Greco L.F., Martinez N., Amstalden M., Thatcher W.W., Santos J.E.P. (2013). Hormonal manipulations in the 5-day timed artificial insemination protocol to optimize estrous cycle synchrony and fertility in dairy heifers. J. Dairy Sci..

[bib8] Martins J.P.N., Policelli R.K., Neuder L.M., Raphael W., Pursley J.R. (2011). Effects of cloprostenol sodium at final prostaglandin F2α of Ovsynch on complete luteolysis and pregnancy per artificial insemination in lactating dairy cows. J. Dairy Sci..

[bib9] Monteiro P.L.J., Nascimento A.B., Pontes G.C.S., Fernandes G.O., Melo L.F., Wiltbank M.C., Sartori R. (2015). Progesterone supplementation after ovulation: Effects on corpus luteum function and on fertility of dairy cows subjected to AI or ET. Theriogenology.

[bib10] Niles A.M., Fricke H.P., Carvalho P.D., Wiltbank M.C., Hernandez L.L., Fricke P.M. (2019). Effect of treatment with human chorionic gonadotropin 7 days after artificial insemination or at the time of embryo transfer on reproductive outcomes in nulliparous Holstein heifers. J. Dairy Sci..

[bib11] NRC (2001).

[bib12] Rizos D., Scully S., Kelly A.K., Ealy A.D., Moros R., Duffy P., Al Naib A., Forde N., Lonergan P. (2012). Effects of human chorionic gonadotrophin administration on Day 5 after oestrus on corpus luteum characteristics, circulating progesterone and conceptus elongation in cattle. Reprod. Fertil. Dev..

[bib13] Sala R.V., Melo L.F., Motta J.C.L., Leffers-Neto L., Carrenho-Sala L.C., Fosado M., Moreno J.F., Baruselli P.S., Wiltbank M.C., García-Guerra A. (2020). Optimization of a 5-day fixed-time embryo transfer (FTET) protocol in heifers I. Manipulation of circulating progesterone through reutilization of intravaginal progesterone devices during FTET. Theriogenology.

[bib14] Schmitt É. J.P., Diaz T., Barros C.M., De La Sota R.L., Drost M., Fredriksson E.W., Staples C.R., Thorner R., Thatcher W.W. (1996). Differential response of the luteal phase and fertility in cattle following ovulation of the first-wave follicle with human chorionic gonadotropin or an agonist of gonadotropin-releasing hormone. J. Anim. Sci..

[bib15] Steichen M.M., Larson J.E. (2019). Effects of supplemental progesterone using a CIDR insert on pregnancy per embryo transfer of dairy heifer recipients of embryos produced in vitro. Anim. Reprod. Sci..

[bib16] Yen S.S., Llerena O., Little B., Pearson O.H. (1968). Disappearance rates of endogenous luteinizing hormone and chorionic gonadotropin in man. J. Clin. Endocrinol. Metab..

